# Effects of the nerve agent VX on hiPSC-derived motor neurons

**DOI:** 10.1007/s00204-024-03708-3

**Published:** 2024-03-30

**Authors:** Catherine Schaefers, Wolfgang Schmeißer, Harald John, Franz Worek, Theo Rein, Simone Rothmiller, Annette Schmidt

**Affiliations:** 1 Bundeswehr Institute of Pharmacology and Toxicology, Neuherbergstr. 11, 80937 Munich, Germany; 2 Max Planck Institute of Psychiatry, Kraepelinstr. 2-10, 80804 Munich, Germany; 3 Institute of Sport Science, University of the Bundeswehr Munich, Werner-Heisenberg-Weg 39, 85577 Neubiberg, Germany

**Keywords:** Apoptosis, qRT-PCR, BCL2A1, CASP10, Neurotoxicity, Nerve agent

## Abstract

Poisoning with the organophosphorus nerve agent VX can be life-threatening due to limitations of the standard therapy with atropine and oximes. To date, the underlying pathomechanism of VX affecting the neuromuscular junction has not been fully elucidated structurally. Results of recent studies investigating the effects of VX were obtained from cells of animal origin or immortalized cell lines limiting their translation to humans. To overcome this limitation, motor neurons (MN) of this study were differentiated from in-house feeder- and integration-free-derived human-induced pluripotent stem cells (hiPSC) by application of standardized and antibiotic-free differentiation media with the aim to mimic human embryogenesis as closely as possible. For testing VX sensitivity, MN were initially exposed once to 400 µM, 600 µM, 800 µM, or 1000 µM VX and cultured for 5 days followed by analysis of changes in viability and neurite outgrowth as well as at the gene and protein level using µLC-ESI MS/HR MS, XTT, IncuCyte, qRT-PCR, and Western Blot. For the first time, VX was shown to trigger neuronal cell death and decline in neurite outgrowth in hiPSC-derived MN in a time- and concentration-dependent manner involving the activation of the intrinsic as well as the extrinsic pathway of apoptosis. Consistent with this, MN morphology and neurite network were altered time and concentration-dependently. Thus, MN represent a valuable tool for further investigation of the pathomechanism after VX exposure. These findings might set the course for the development of a promising human neuromuscular test model and patient-specific therapies in the future.

## Introduction

The highly toxic chemical warfare nerve agent VX is structurally related to widely used pesticides and belongs to the group of organophosphorus compounds (OPC) (Wang et al. 2008; Munro 1994). Although the development, production, stockpiling, and use of chemical warfare agents has been banned by the Chemical Weapons Convention since 1997 and is supervised by the Organisation for Prohibition of Chemical Weapons (OPCW), the threat still remains as recent deployments in Syria and assassinations in Japan or Malaysia demonstrate (Organisation for the Prohibition of Chemical Weapons 2022; Nakagawa and Tu 2018; Tu 2020; John et al. 2018). By acute exposure to nerve agents like VX, clinical signs of poisoning, such as bradycardia, salivation, lacrimation, tremor, seizures, and finally respiratory failure, are induced due to the irreversible inhibition of acetylcholineesterase (AChE). AChE inhibition leads to an excess of acetylcholine (ACh) in the synaptic cleft and over-stimulation of nicotinergic and muscarinergic ACh-receptors (AChR) (Thiermann et al. 2016). Great efforts have been made to develop treatments concerning reactivation of AChE, like oximes, or competitive antagonism of muscarinergic AChR, e.g., atropine, or catalytic bioscavengers that hydrolyse or bind nerve agents (Worek et al. 2014). Unfortunately, these therapeutic approaches show insufficient efficacy in some poisonings and long-term, non-cholinergic effects, such as organophosphorus ester-induced chronic neurotoxicity (OPICN) or organophosphorus ester-induced delayed neurotoxicity (OPIDN), as well as dysfunctions at the neuromuscular synapses could not be addressed so far (Abou-Donia 2003; Thiermann et al. 2010, 2013). As the conduction of clinical trials is unacceptable from an ethical point of view, most of the results were obtained from animal experiments or cells of animal origin in awareness that translation of findings from animal studies to humans is only possible to a limited extent (Thiermann et al. 2016; Bloch-Shilderman et al. 2018; Heppner and Fiekers 1992; Tenn et al. 2012). For the further development and improvement of therapeutic strategies, it is crucial to investigate the mechanisms that occur at the human neuromuscular junction upon nerve agent exposure (Thiermann et al. 2010). This points out the need of a human neuromuscular test model consisting of motor neurons and skeletal muscle cells.

Recent studies investigating the effects of VX exposure in neurons used immortalized neuronal cell lines (Kanjilal et al. 2014), being genetically modified and thereby may depict an altered responsiveness to stimuli (Kaur and Dufour 2012) or cells of human embryonic stem cells (hESC) origin (Gao et al. 2013) that are controversial from an ethical point of view. Moreover, culture media supplemented with antibiotics which is commonly used for maintenance of cells (Kanjilal et al. 2014; Gao et al. 2013), may induce changes in gene expression and regulation (Ryu et al. 2017) and might impair neuronal cell survival and regeneration (Lindsey and Townes-Anderson 2018). Thus, the cell cultures used so far are potentially unsuitable for implementation in a human neuronal test model.

In this study, human mature motor neurons (MN) were differentiated from in-house generated and verified human-induced pluripotent stem cells (hiPSC) to omit the use of ethically controversial hESC. Furthermore, a human-like developmental process similar to embryogenesis was guaranteed by avoiding xenogenic materials such as viruses for transfection or antibiotics in cell culture. Standardized cell culture media were used for the generation of hiPSC as well as differentiation of MN to ensure the reproducibility and robustness of the applied protocol. Thus, the hiPSC-differentiated and antibiotic-free maintained MN are best suited for implementation into a neuromuscular model in terms of translation.

So far, there are no data concerning the effects of VX on MN derived from hiPSC. To evaluate the hiPSC-differentiated MN for the potential application in a human neuronal test model, MN were treated with VX followed by investigations on neurite outgrowth and changes at the gene and protein level. This study provides new insight into the pathomechanism of VX in MN and might set the course for the development of a human neuromuscular test model and new therapeutic approaches.

## Materials and methods

### Generation of MN from hiPSC

MN were differentiated from neuronal progenitor cells (NPC) which were derived from hiPSC as described before by Schaefers et al. (2022). In brief, hiPSC were generated from human neonatal dermal fibroblasts (ATCC, Manassas, Virginia, USA) by episomal nucleotransfection with three plasmids (pCXLE-hOCT3/4 shp53-F, pCXLE-hSK, pCXLE-hUL; Addgene, Watertown, Massachusetts, USA) encoding reprogramming factors OCT3/4, SOX2, KLF4, and L-MYC. hiPSC were maintained for 30 days in mTeSR™1 (Stemcell Technologies, Vancouver, Canada) with a change of media every other day and passaging every 5–7 days using Gentle Cell Dissociation Reagent (Stemcell Technologies, Vancouver, Canada) onto Corning^®^ Matrigel^®^ (Corning, New York, New York, USA) pre-coated 6-well plates. In the following, hiPSC were differentiated into NPC using STEMDiff™ SMADi Neural Induction Kit (Stemcell Technologies, Vancouver, Canada) with passaging onto pre-coated poly-L-ornithine/laminin plates (both Sigma-Aldrich, St. Louis, Missouri, USA) every 5–7 days using STEMDiff™ Neural Rosette Selection Reagent (Stemcell Technologies, Vancouver, Canada). NPC were expanded using STEMDiff™ Neural Progenitor Medium (NPM, Stemcell Technologies, Vancouver, Canada) for 21 days and passaged with StemPro™ Accutase™ Cell Dissociation Reagent (Thermo Fisher, Waltham, Massachusetts, USA). For generation of mature neurons, NPC were exposed to STEMDiff™ Neuron Differentiation Medium for 7 days followed by culturing in STEMDiff™ Neuron Maturation Medium (NMM) for 21 days (both Stemcell Technologies, Vancouver, Canada). Cells were passaged onto pre-coated poly-L-ornithine/laminin plates every 5–7 days using StemPro™ Accutase™ Cell Dissociation Reagent (Thermo Fisher, Waltham, Massachusetts, USA). The identity of hiPSC, NPC, and mature neurons was confirmed by the expression of stage-specific markers by immunofluorescence, gene expression, flow cytometry, and Western Blot analysis as shown by Schaefers et al. (2022).

### Exposure to different concentrations of VX

VX (CAS no. 50782–69-9, > 98% ^1^H-NMR) was provided by the German Ministry of Defence. Prior to the experiments, VX was freshly pre-diluted in acetonitrile (ACN, Merck, Darmstadt, Germany) to a final concentration of 37.4 mM VX and stored at room temperature for further use. For the experiments, pre-diluted VX was diluted in NMM for MN and NPM for NPC. The final concentration of ACN was 2.67% (v/v) in the experiments with no decrease in cell viability observed. Working with higher VX concentrations and correspondingly lower solvent concentrations was not feasible due to the hazard potential of VX and in-house safety regulations.

MN and NPC were passaged onto pre-coated poly-l-ornithine/laminin 24-well plates (Greiner AG, Kremsmünster, Austria) at a density of 140,000 cells per well in NMM for MN or NPM for NPC using StemPro™ Accutase™ Cell Dissociation Reagent. Briefly, cells were incubated for 5 min at 37 °C in humidified atmosphere containing 5% (v/v) CO_2_, followed by centrifugation for 5 min at 296 × g.

After incubation for 48 h at 37 °C in humidified atmosphere containing 5% (v/v) CO_2_, cells were exposed to a final concentration of 400 µM, 600 µM, 800 µM, 1000 µM VX or ACN alone (solvent control) in NMM including medium control (3 wells, each). The VX concentrations used were higher than those of previously published experiments (Gao et al. 2013) as exposure to lower VX concentrations revealed no changes in cell viability (data not shown). A relevant decrease in VX concentration due to hydrolysis is unlikely as VX has a period of half-change for hydrolysis of 1,000 h (pH 7.0) (John et al. 2020). These findings provided the basis for the experiments in the present study.

### Cell lysis and preparation for µLC-ESI MS/HR MS analysis

After exposure of MN to 400 µM VX, 1000 µM VX or ACN alone (solvent control) for 5 days, cells were detached by cell scraper and washed three times with PBS (Gibco, Karlsruhe, Germany) by careful resuspension after centrifugation for 10 min at 211×g at 4 °C. According to Lüling et al. (2021), cell lysis was performed as described below. After discarding the supernatant, the pellet was lysed under repeated resuspension using lysis buffer (7 M urea, 2 M thiourea, 4% w/v CHAPS [all Sigma-Aldrich, St. Louis, Missouri, USA] and 10 μL/mL protease inhibitor mix [GE Healthcare, Freiburg, Germany] in ultra-pure water) and was kept on ice. Sonication was performed four times at 10 s, 0.3 interval, and 30% intensity with ultrasonic homogeniser (Bandelin electronic, Berlin, Germany). After adding 10 µL/mL nuclease mix (GE Healthcare, Freiburg, Germany), the protein lysate was incubated for 45 min at room temperature followed by centrifugation for 10 min at 21,130 × g at 4 °C. After determination of the protein concentration using Qubit™ Protein Assay Kit with Qubit 4 Fluorometer (both Thermo Fisher, Waltham, Massachusetts, USA), the supernatant was aliquoted in Protein LoBind^®^ 0.5 mL tubes (Eppendorf AG, Hamburg, Germany) and stored at − 80 °C until further use.

Enzymatic proteolysis of protein lysate was performed based on the protocol of Kranawetvogl et al. (2018). In brief, 200 µL protein lysate (10 mg/mL) was transferred into an ultrafiltration (UF) device (Amicon Ultra-0.5 Centrifugal Filter Units, molecular weight cut-off 10 kDa; Merck, Darmstadt, Germany), centrifuged for 10 min at 10,270×g at 15 °C and washed three times by UF (10,270×g, 15 °C, 5 min) using 350 µL NH_4_HCO_3_ buffer (50 mM ammoniumbicarbonate [ultra-grade ≥ 99.5% Fluka, Buchs, Switzerland] in water [LC–MS grade, Merck, Darmstadt, Germany]), each. For enzymatic cleavage, 100 µL pronase solution (60 mg/mL pronase [Roche, Mannheim, Germany] in NH_4_HCO_3_ buffer) and 200 µL NH_4_HCO_3_ buffer were added to the retentate and incubated for 2 h at 42 °C under gentle shaking. Afterwards, residual pronase and larger polypeptides were separated by centrifugation for 10 min at 10,270×g at 15 °C. The filtrate was diluted 1:3 with a solution of threefold deuterated atropine (d_3_-Atr, 3 ng/mL d_3_-Atr [CDN Isotopes, Pointe-Claire, Quebec, Canada] in 0.5% v/v formic acid [≥ 98%, Sigma-Aldrich, St. Louis, Missouri, USA]) prior to micro liquid chromatography–electrospray ionization high-resolution tandem-mass spectrometry (µLC–ESI MS/HR MS) analysis in product ion scan (PIS) mode.

### µLC–ESI MS/HR MS PIS analysis of phosphonylated tyrosines

For monitoring tyrosine residues phosphonylated by an ethylmethylphosphonyl (EMP) moiety derived from the pronase-catalyzed proteolysis of VX-modified proteins, μLC-ESI MS/HR MS measurements were performed according to Kranawetvogl et al. (2016).

Therefore, a microLC 200 pump (Eksigent Technologies LLC, Dublin, CA, USA) on-line coupled to a TripleTOF 5600 + mass spectrometer (TT5600 + , SCIEX, Darmstadt, Germany) was used as described recently (Kranawetvogl et al. 2016; Schmeißer et al. 2022).

Chromatographic separation of 20 µL sample volume was performed on an Acquity HSS T3 column (50 × 1.0 mm I.D., 1.8 μm, 100 Å, Waters, Eschborn, Germany) protected by a precolumn (Security Guard™ Ultra Cartiges UHPLC C18 peptide 2.1 mm I.D., Phenomenex, Aschaffenburg, Germany) at 65 °C. For gradient elution, a mobile phase consisting of solvent A (0.05% v/v FA) and solvent B (ACN/H_2_O 80:20 v/v, 0.05% v/v FA) was applied at a total flow of 30 μL/min as follows: t [min]/B [%]: 0/4, 3/4, 15/39, 16/95, 24/95, 25/4, and 30/4. The µLC system was controlled by the Eksigent 4.2 (Eksigent Technologies LLC) software.

Product ions of single protonated phosphonylated tyrosine residues (Tyr-EMP, [M + H]^+^, *m/z* 288.1) and d_3_-Atr ([M + H]^+^, *m/z* 293.2, used as internal standard, IS) obtained after collision-induced dissociation (CID) were monitored in a mass range from *m/z* 50 to *m/z* 650 with an accumulation time of 300 ms, each. For detection, the following MS parameters were used: collision energy 25 V (Tyr-EMP) and 42 V (d_3_-Atr), declustering potential 60 V, curtain gas 3.45 × 10^5^ Pa (50 psi), turbo ion spray gas 3.45 × 10^5^ Pa (50 psi), heater gas 2.76 × 10^5^ Pa (40 psi), ion spray voltage floating 4500 V, temperature 200 °C, accumulation time 300 ms, ion release delay 67 ms, and ion release width 25 ms.

For data acquisition, processing, and analysis, the Analyst TF 1.7.1, PeakView 2.1 (SCIEX) and GraphPad Prism version 5.04 (GraphPad Software, La Jolla, CA, USA) software were used.

### Determination of cell viability after VX exposure

NPC and MN were plated at 140,000 cells per well onto pre-coated poly-l-ornithine/laminin 24-well plates and exposed to 400 µM VX, 600 µM VX, 800 µM VX, 1000 µM VX or ACN alone (solvent control) in NMM (3 wells each) as described above. After incubation for 5 days, cells were washed once with PBS. XTT staining solution (Sigma-Aldrich, St. Louis, USA) was prepared freshly by mixing 100 µL coupling reagent and 5 mL XTT labelling reagent. After incubating the cells for 3 h with 400 µL NMM and 200 µL XTT staining solution in a humidified atmosphere at 37 °C containing 5% (v/v) CO_2_, the absorbance was quantified at 450 nm and a reference wavelength at 630 nm. After elimination of background absorbance determined in the wells containing only NMM, the viability was normalized to solvent controls. The experiment was carried out three times independently (biological replicates) in three technical replicates per concentration.

### Confluence detection by the IncuCyte^®^

MN were exposed to VX as described above. The 24-well plates were subsequently placed in the IncuCyte^®^ S3 system (Sartorius, Göttingen, Germany) and phase-contrast images were captured every hour to investigate effects of VX over a period of 5 days. The confluence of cells was determined by measuring the occupied area and is given in percentage of the initial confluence at the first image. The experiment was carried out in technical triplicates and three times independently.

### Detection of neurite network after VX exposure

400,000 MN were seeded onto pre-coated poly-l-ornithine/laminin 1-well Nunc™ Lab-Tek™ II Chamber Slide™ (Thermo Fisher, Waltham, Massachusetts, USA) as described above and were incubated for 48 h at 37 °C in a humidified atmosphere containing 5% (v/v) CO_2_. Cells were exposed to 400 µM, 600 µM, 800 µM, and 1000 µM VX or ACN alone (solvent control) and transferred to the IncuCyte^®^ S3 system (Sartorius, Göttingen, Germany). Phase-contrast images of the cells were acquired before and after exposure for 5 days, every 6 h. Changes in neurite outgrowth were determined using the IncuCyte^®^ NeuroTrack Software Module (Sartorius, Göttingen, Germany). The experiment was performed three times independently.

### Changes in gene expression by qRT-PCR

After 5 days of exposure to 400 µM, 600 µM, 800 µM, 1000 µM VX or solvent control, MN were detached with a cell scraper (Greiner AG, Kremsmünster, Austria) into culture medium. For total RNA extraction using RNeasy Mini Kit (Qiagen, Hilden, Germany), cells were washed three times with PBS (Gibco, Karlsruhe, Germany) with centrifugation for 3 min at 211×g. Subsequently, cells were lysed under repeated resuspension in 400 µL buffer RLT. According to the manufacturer’s protocol, the lysate was added to a QIAshredder spin column (Qiagen, Hilden, Germany) and centrifuged for 2 min at 21,135×g. The eluate was mixed with 400 µL of 70% (v/v) ethanol. 700 µL of the mixture was transferred to a spin column and centrifuged for 30 s at 13,250×g. The column containing the bound RNA was washed once with 700 µL buffer RW1 (30 s, 13,250×g) and twice with 500 µL buffer RPE (2 min, 21,135×g). Residual buffer was eliminated by spinning for 1 min at 21,135×g. RNA elution was performed with 30 µL nuclease-free water (Qiagen, Hilden, Germany) by centrifugation for 1 min at 21,135×g into a Biopur^®^ 1.5 mL tube (Eppendorf AG, Hamburg, Germany).

RNA concentration was assessed by the NanoQuant Plate™ with plate reader Infinite M200 Pro (Tecan Group AG, Männedorf, Switzerland). For cDNA-synthesis, RT^2^ First-Strand Kit (Qiagen, Hilden, Germany) was used according to the manufacturer’s instructions and was conducted with 500 ng RNA per qPCR-plate. In brief, a mix of 2 µL GE buffer, RNA and RNase-free water was prepared yielding a total volume of 10 µL. For elimination of genomic DNA, the mix was incubated for 5 min at 42 °C, followed by cooling on ice for at least 1 min. 4 µL 5 × buffer BC3, 1 µL control P2, 2 µL RE3 reverse transcriptase mix and 3 µL RNase-free water were mixed carefully. The total volume was added to the genomic DNA elimination mix for subsequent reverse transcription by incubation for 15 min at 42 °C followed by incubation for 5 min at 95 °C. All incubation steps were carried out with the Mastercycler^®^ nexus GX2 (Eppendorf AG, Hamburg, Germany). After adding 91 µL nuclease-free water, the prepared cDNA was stored at − 20 °C for a maximum of seven days.

According to the manufacturer’s protocol, 102 µL of cDNA preparation was mixed with 1,248 µL RNase-free water. The mixture was placed in the Freedom Evo automated pipetting machine controlled by EVOware™ Standard Software (Tecan Group AG, Männedorf, Switzerland). Before and after each following pipetting step, the tips of Freedom Evo were extensively treated with 7% (v/v) sodium hypochlorite (Carl Roth, Karlsruhe, Germany) in ultra-pure water followed by washing several times with ultra-pure water. 1,350 µL of 2 × RT^2^ SYBR^®^ Green ROX qPCR Mastermix (Qiagen, Hilden, Germany) was added to the cDNA dilution followed by gentle mixing by inversion. 25 µL of this mix was dispensed into each well of following RT^2^ Profiler™ PCR Arrays: Human Apoptosis, Human Neurotoxicity, Human DNA Damage Signalling Pathway (all Qiagen, Hilden, Germany). After sealing each plate, qRT-PCR analysis was carried out using Eppendorf Mastercycler^®^ epgradient S realplex^2^ with Mastercycler ep realplex software (both Eppendorf AG, Hamburg, Germany) by activating polymerase for 10 min at 95 °C followed by 40 cycles of 15 s at 95 °C and 1 min at 60 °C. To better compare the experiments, the threshold was set manually to 200 and drift correction was enabled.

To analyse the data via GeneGlobe (http://www.qiagen.com/geneglobe), C_t_ values were exported, and the following settings were defined: C_t_ cut-off (35), fold regulation cut-off (2) and p value cut-off (0.05). The housekeeping gene RPLP0 was taken for normalization. Fold regulations were calculated using the 2^(−ΔΔCt)^ formula and p values were based on an unpaired Student’s t test. The experiment was performed three times independently.

### Western blot

MN, exposed to 400 µM, 600 µM, 800 µM, 1000 µM VX or ACN alone (solvent control) for 5 days, were collected by cell scraper and were washed three times with PBS (Gibco, Karlsruhe, Germany) by careful resuspension after centrifugation for 3 min at 211 × g. Subsequently, neurons were lysed under repeated suspension with 400 µL Tris–EDTA-Triton X-100 extraction buffer (6.25 mM TRIS, 12.5 mM NaCl, 2.5 mM EDTA, 1.5% (v/v) Triton X-100 [all Sigma-Aldrich, St. Louis, Missouri, USA], one Complete Mini Inhibitor Cocktail and one PhosSTOP [both Roche, Basel, Switzerland] in 10 mL ultra-pure water) and were incubated on ice for 15 min as described before by Schaefers et al. (2022). In brief, disruption of cells was achieved using ultrasonic homogeniser (Bandelin electronic, Berlin, Germany) three times for 10 s, 0.3 interval, and 30% intensity, followed by incubation on ice for 1 h, with vortexing every 15 min. After centrifugation for 10 min at 17,186 × g at 4 °C, the protein concentration was determined from the supernatant using Qubit™ Protein Assay Kit with Qubit 4 Fluorometer (both Thermo Fisher, Waltham, Massachusetts, USA) according to the manufacturer’s protocols. The lysate was aliquoted and stored at − 20 °C.

For each Western Blot, duplicates of 40 µg protein were denatured by mixing with 8 µL loading buffer (60% v/v of 4 × Protein Sample Loading Buffer [LI-COR Biosciences, Lincoln, Nebraska, USA] and 3.12% w/v DTT [Sigma-Aldrich, St. Louis, Missouri, USA] in ultra-pure water) and ultra-pure water to a total volume of 25 µL per sample followed by subsequent incubation for 5 min at 95 °C. 3 µL of Chameleon™ Duo Pre-stained Protein Ladder (LI-COR Biosciences, Lincoln, Nebraska, USA) and the samples were loaded on two NuPAGE™ 4–12% Bis–Tris gels 1.0 mm × 10 wells with NuPAGE™ MES SDS Running Buffer (both Novex by Thermo Fisher Scientific, Waltham, USA). Protein separation of both gels (gel I and II) was performed in the same Mini Gel Tank (Invitrogen by Thermo Fisher Scientific, Waltham, USA) for 50 min at 200 V. One gel (gel I) was then used for transfer of proteins using iBlot™ Transfer Stack PVDF (0.2 µm pore size) with iBlot™ 2 Gel Transfer Device (both Thermo Fisher, Waltham, Massachusetts, USA) for 7 min (1 min 20 V, 4 min 23 V, 2 min 25 V). The membrane was rinsed once in ultra-pure water, activated with methanol (Sigma-Aldrich, St. Louis, Missouri, USA) by rinsing for 30 s and washing twice with ultra-pure water. Subsequently, the membrane was blocked in Intercept^®^ (PBS) Blocking Buffer (LI-COR Biosciences, Lincoln, Nebraska, USA) for 1 h followed by incubation overnight at 4 °C with the following primary antibody in antibody diluent (Intercept^®^ (PBS) Blocking Buffer with 0.2% v/v Tween^®^ 20 [Sigma-Aldrich, St. Louis, Missouri, USA]): Recombinant Rabbit Anti-Caspase-10 (ab177475 abcam, Cambridge, UK) 1:5,000. After washing twice for 10 min with washing buffer (PBS with 0.1% v/v Tween^®^ 20), the membrane was incubated with secondary antibody for 90 min under light exclusion with IRDye^®^ 800CW Goat Anti-Rabbit (LI-COR Biosciences, Lincoln, Nebraska, USA) 1:8,000 in antibody diluent. The membrane was washed again two times for 10 min with washing buffer, rinsed twice with ultra-pure water, and was imaged with the Odyssey CLx imaging systems. For total protein normalization, the other gel (gel II) was stained using Coomassie (0.1% w/v Coomassie Blue R-350 [Cytiva, Marlborough, Massachusetts, USA], 30% v/v methanol, and 10% v/v acetic acid [both Sigma-Aldrich, St. Louis, Missouri, USA] in ultra-pure water) for 45 min followed by washing with ultra-pure water three times for 1 h, each, and additional washing overnight. Prior to imaging the gel using Odyssey CLx with Empiria Studio software (LI-COR Biosciences, Lincoln, Nebraska, USA), gel II was washed with ultra-pure water for 5 min. This method was used to overcome the problem of differential expression of b-actin after VX exposure as observed in previous experiments (data not shown) and in the awareness that a discrepancy may occur due to differences in transfer efficiency when comparing stained gel and probed membrane. Since differences in the molecular weight and not the variability between lanes lead to inconsistencies in transfer efficiency (Eaton et al. 2013), commercially produced transfer packs, such as iBlot™ Transfer Stack PVDF used here, should reduce this variation to a minimum. Furthermore, every experiment was performed in the same electrophoretic tank and with the same loading buffer, prefabricated gels, membranes, and running buffer of the same batch to account for and minimize variations.

The Empiria Studio^®^ software version 2.2 (https://www.licor.com/bio/empiria-studio/resources) was used for densiometric analysis. Signals of caspase-10 were normalized for total protein signal of the entire lane followed by analysis of each replicate sample. The software RStudio (version 4.0.3 [2020–10-10], RStudio Inc., Boston, USA) was used to illustrate the results. The experiment was carried out three times independently.

### Statistics

Except for MS data, RStudio (version 1.2.1335 with R version 4.0.3 [2020–10–10]) was used for presentation and analysis of all data (RStudio Inc., Boston, USA). Cells exposed to VX were compared to solvent control by the unpaired two-sample Wilcoxon test. *P* values < 0.05 were considered statistically significant. Means and smoothening function with 99% confidence intervals are displayed for representation of IncuCyte^®^ results of confluence and neurite length acquired over time. Tukey boxplots with illustrated single data points were used for analysis of cell viability, confluence, and neurite length after VX exposure for 120 h. qRT-PCR results are shown as heatmaps only for fold regulations ≥ 2.0 or ≤  − 2.0 in combination with *p* < 0.05 and as volcano plots including all data.

## Results

### VX modifies cellular proteins in MN

**Fig. 1:**
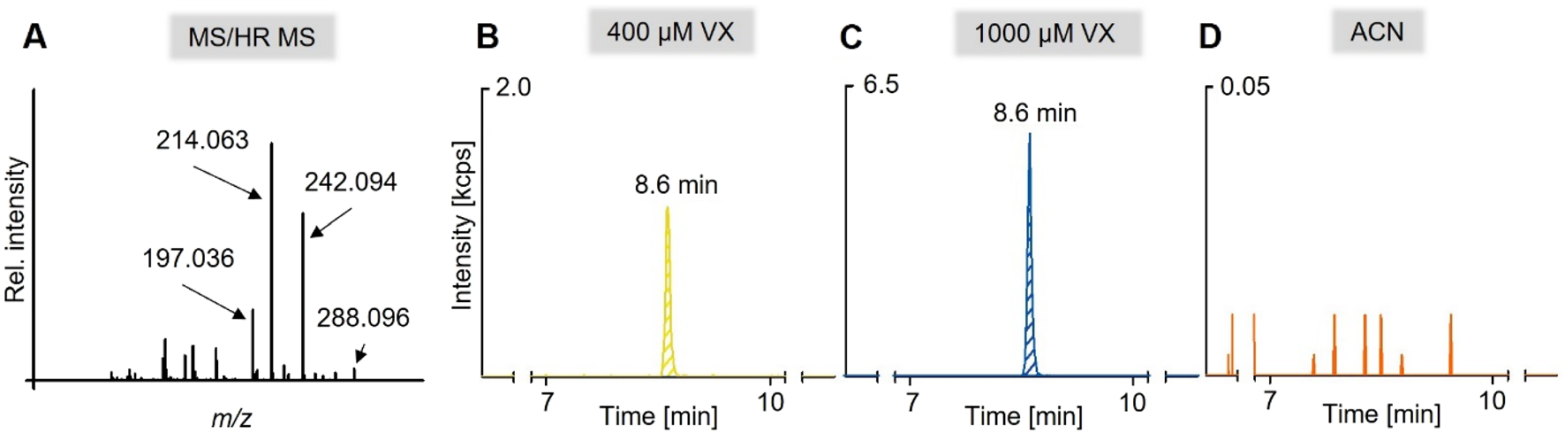
Mass spectrometric detection of VX-derived tyrosine adducts, Tyr(-EMP). The MS/HR MS spectrum of Tyr(-EMP) (*m/z* 288.096) (**A**) was extracted from the chromatographic peak of 1000 µM VX at 8.6 min **(C)**. The most prominent structure confirming product ions are labelled. μLC-ESI MS/HR MS analysis of phosphonylated tyrosines Tyr(-EMP) derived from 400 µM VX (**B**), 1000 µM VX (**C**), and solvent control (ACN) (**D**) exposed MN after lysis and pronase-catalyzed proteolysis. Traces represent extracted ion chromatogram of the most intense product ion of Tyr(-EMP) (*m/*z 214.063 ± 0.005)

As evidence that the VX concentrations used in this study are sufficient to reach and modify cellular proteins, phosphonylated tyrosine residues Tyr(-EMP) obtained after proteolysis with pronase are well-established biomarkers (Kranawetvogl et al. 2016; John et al. 2023). Following this approach, the well-known diagnostic ions at *m/z* 214.063, *m/z* 242.094, and *m/z* 197.036, representing the three most intense product ions of protonated Tyr(-EMP) (*m/z* 288.096), were monitored by μLC-ESI MS/HR MS (PIS) (Fig. 1A). All three diagnostic ions were detected, which allowed an unambiguous identification of Tyr(-EMP). The extracted ion chromatogram of the most intense product ion at *m/z* 214.063 ± 0.005 showed a narrow peak at a retention time (*t*_R_) of 8.6 min in 400 µM VX (Fig. 1B) and 1000 µM VX (Fig. 1C) cell lysate samples. No peak and no interferences were detected in the solvent control, showing highest selectivity of the method (Fig. 1D). The peak area of the 1000 µM VX sample was 2.23 times higher when compared to 400 µM VX, corresponding to a concentration factor of 2.5. This demonstrated a concentration-dependent interaction of VX with protein structures in MN.

### VX decreases neuronal viability

**Fig. 2:**
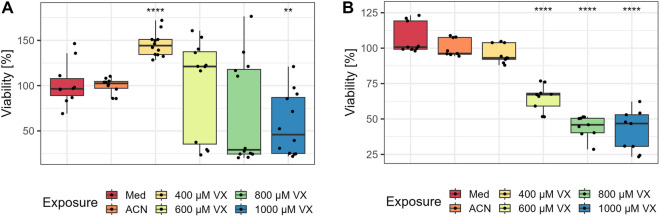
Changes in viability. After 5 days of exposure to 400 µM, 600 µM, 800 µM, 1000 µM VX, medium (Med) or solvent (ACN) control, the viability of mature neurons (**A**) and NPC (**B**) was tested using the XTT assay. The percentage of viable cells (*n* = triplicates per group from three independent experiments) and the significance of each group compared to corresponding solvent controls are illustrated. Data are represented as Tukey boxplots. **p < 0.01, ****p < 0.0001

To detect effects of VX on the viability of MN, the XTT assay was performed after 120 h of exposure to 400 µM, 600 µM, 800 µM, 1000 µM VX, medium (Med) or solvent (ACN) control. In comparison to solvent control, MN exposed to 400 µM VX showed significantly higher viability (145.44 ± 13.25%), while MN exposed to 1000 µM VX exhibited a significant loss of viability (57.12 ± 34.73%). MN exposed to 600 µM (99.17 ± 53.17%) or 800 µM (69.64 ± 57.69%) VX did not reveal significant changes in viability (Fig. 2A). In contrast, using the same assay conditions, NPC revealed a significant loss of viability after exposure to 600 µM (64.89 ± 9.21%), 800 µM (44.72 ± 7.59%), or 1000 µM (41.49 ± 14.34%) VX compared to solvent control. No differences in viability were observed in NPC exposed to 400 µM (95.83 ± 6.54%) VX (Fig. 2B). Both, MN (Fig. 2A) and NPC (Fig. 2B) exposed to solvent control did not show viability differences in comparison to medium control.

### Decreased confluence and changes in morphology

**Fig. 3:**
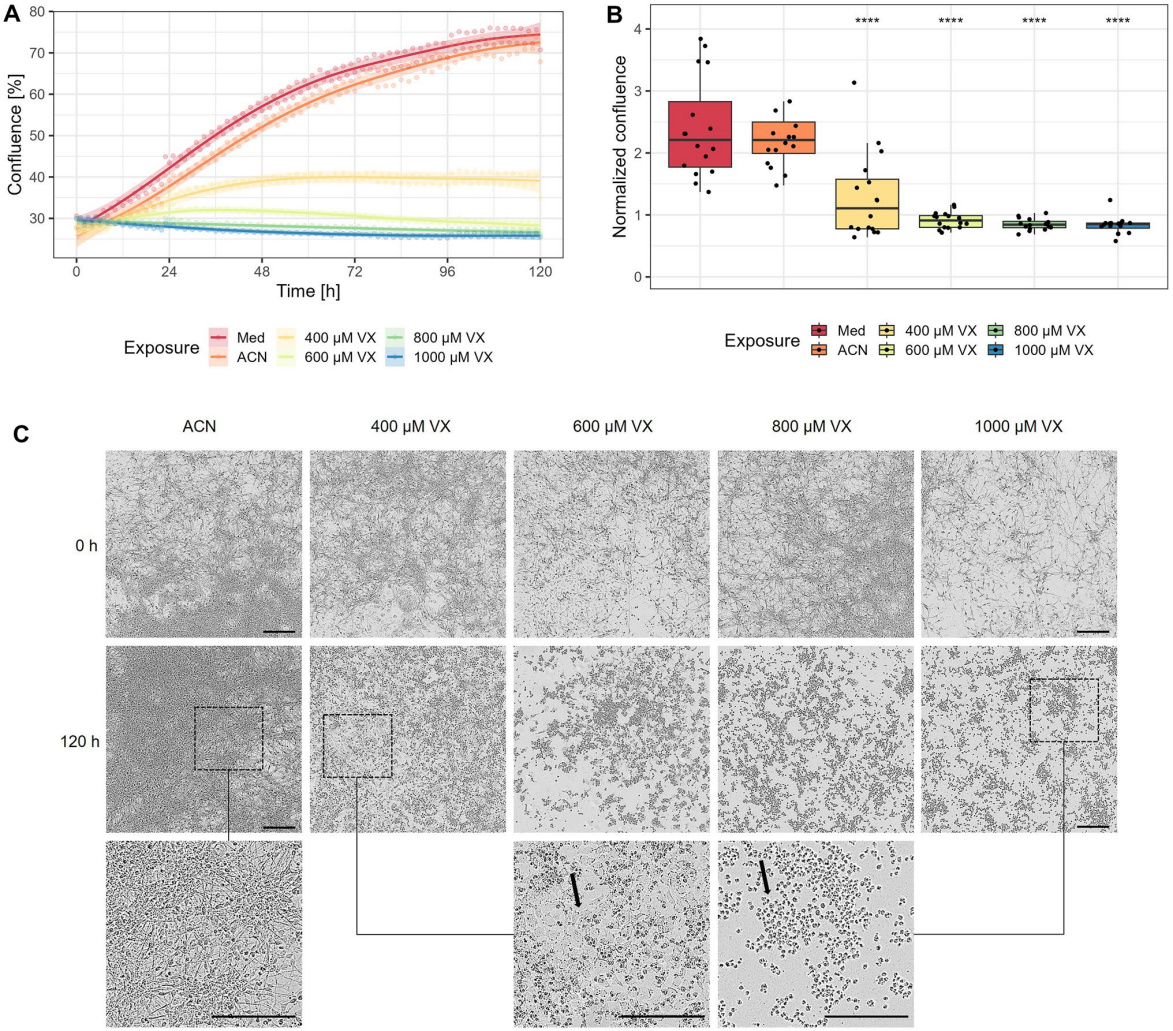
Changes in confluence and morphology of MN. **A** After exposure to 400 µM, 600 µM, 800 µM, 1000 µM VX, medium or solvent (ACN) control, the percentage of confluence was determined by the area covered by MN using the IncuCyte^®^ microscope every hour in a total period of 120 h. Results are illustrated as means and trend with 99% confidence interval (*n* = three independent experiments). **B** Confluence after 120 h of exposure to 400 µM, 600 µM, 800 µM, 1000 µM VX, medium (Med) or solvent (ACN) control normalized to confluence before exposure (0 h). The significance of each group compared to the corresponding solvent controls is presented (*n* = three independent experiments). Data are represented as Tukey box plots. ****p < 0.0001. **C** Representative images from MN before (0 h) and after (120 h) exposure to solvent control (ACN), 400 µM, 600 µM, 800 µM, or 1000 µM VX. For better illustration, images of MN after exposure to solvent control (ACN; left), 400 µM (middle) and 1000 µM (right) VX are additionally displayed with 2.6 × magnification. Black arrows indicate changes in cell-to-cell interactions. Scale bars, 200 µm

To measure changes in cellular growth and observe differences in the morphology of MN after exposure to 400 µM, 600 µM, 800 µM, 1000 µM VX, medium or solvent control over 120 h, the area occupied by cells was determined by imaging every hour using the IncuCyte^®^ system (Fig. 3). An increase in confluence was observed in MN exposed to medium, from 30.62 ± 11.07% (0 h) to 70.72 ± 9.92% (120 h), and solvent control from 27.73 ± 10.23% (0 h) to 67.84 ± 12.06% (120 h). 400 µM VX-exposed cells tended to increase in confluence from 27.58 ± 8.74% (0 h) to 41.47 ± 20.45% (99 h) followed by a decrease to 34.87 ± 20.21% (120 h) over time. To a lower extent, this progression was also observed for MN exposed to 600 µM VX, from 29.18 ± 7.56% (0 h) to 33.42 ± 8.50% (23 h) followed by a decrease to 26.96 ± 7.73% (120 h). In comparison, exposure to 800 µM VX tended to induce a decrease in confluence from 29.97 ± 6.76% (0 h) to 26.15 ± 6.49% (120 h) as well as exposure to 1000 µM VX from 29.99 ± 7.67% (0 h) to 25.51 ± 7.41% (120 h) over time (Fig. 3A). For better comparability of confluence after 120 h of exposure, the values were normalized to the initial value, whereby an increase is indicated by values above 1 and a decrease by values below 1. At all VX concentrations, MN showed a significantly lower confluence in comparison to solvent control, whereas confluence of MN exposed to medium control remained without significant differences. A trend for an increase in confluence was recorded for MN exposed to 400 µM VX (1.29 ± 0.69) whereas a trend for decreasing confluence was observed for MN exposed to 600 µM (0.92 ± 0.13), 800 µM (0.85 ± 0.09), or 1000 µM (0.83 ± 0.14) VX (Fig. 3B). Images from cells acquired before exposure and 120 h after exposure to 400 µM, 600 µM, 800 µM, and 1000 µM VX or solvent control demonstrated changes in morphology. Before exposure, MN appeared as a monolayer-forming cluster of cells with intact cell membrane and cell-to-cell interactions. In the solvent control, an increase in these cells was obtained after 120 h. In comparison, a cell shrinkage and aggregates of floating apoptotic-like bodies were detected after exposure to all VX concentrations. Potentially viable cells with cell-to-cell interactions, but enlarged and flattened appearance, remained after exposure to 400 µM or 600 µM VX which were not detectable after exposure to 800 µM or 1000 µM VX (Fig. 3C).

### Changes in neurite network and decrease in neurite length

**Fig. 4:**
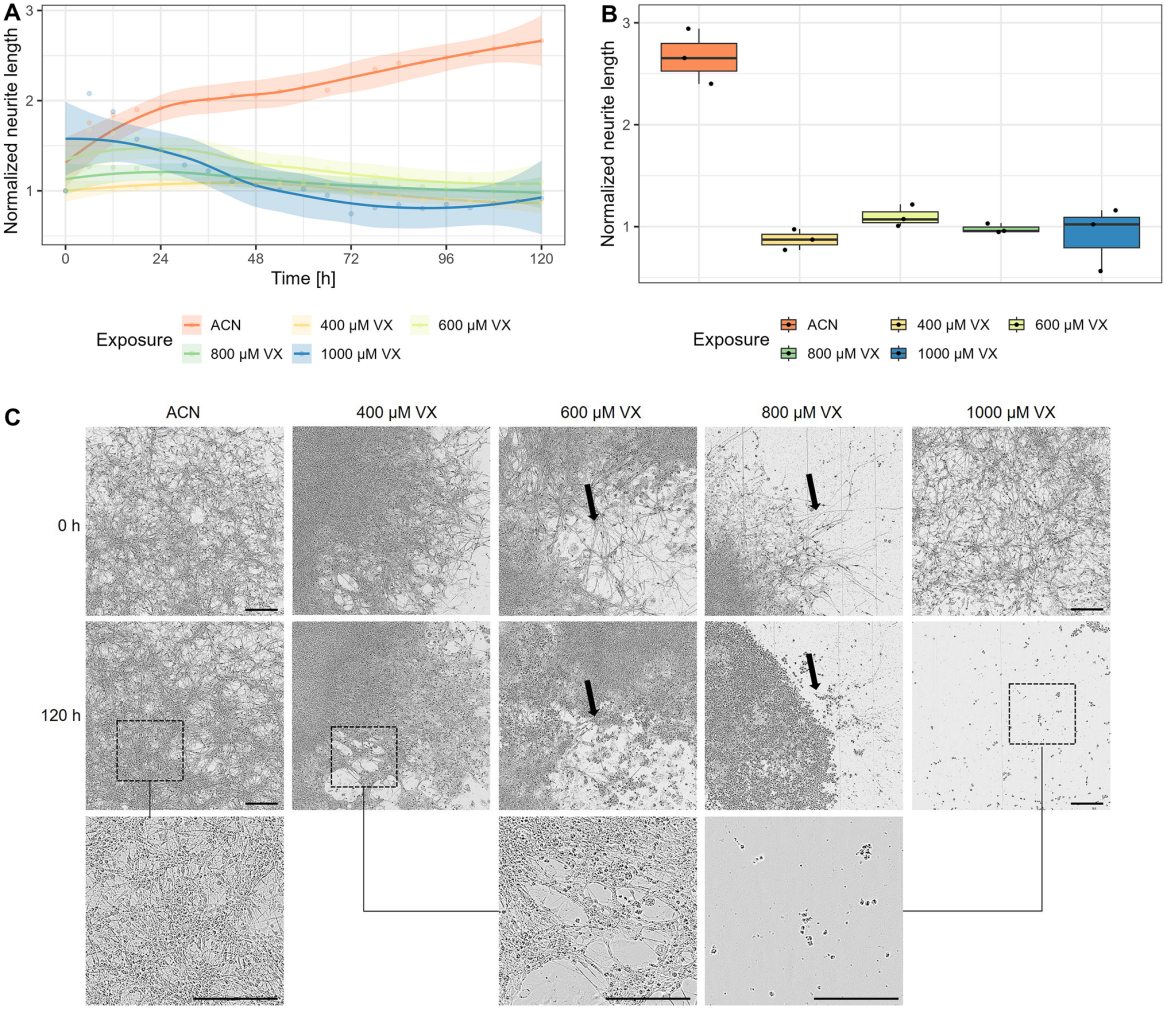
Changes in neurite network of MN. **A** The neurite length of MN, exposed to 400 µM, 600 µM, 800 µM, 1000 µM VX or solvent control (ACN), was determined every 6 h over a period of 120 h using the NeuroTrack software of the IncuCyte^®^ microscope. Results are displayed as means ± SD and trend with 99% confidence interval (*n* = three biological replicates). **B** Illustration of changes in neurite length after 120 h of exposure to 400 µM, 600 µM, 800 µM, 1000 µM VX or solvent control (ACN) normalized to neurite length before exposure (0 h). Data are represented as Tukey box plots (*n* = three biological replicates). **C** Representative images of neurite network before (0 h) and after exposure (120 h) to solvent control (ACN), 400 µM, 600 µM, 800 µM, or 1000 µM VX. Black arrows indicate changes in neurite network. For better illustration, images of MN after exposure to solvent control (ACN; left), 400 µM (middle), or 1000 µM (right) VX with 2.6 × magnification are additionally displayed. Scale bars, 200 µm

To determine effects of VX on MN neurite outgrowth, the neurite length was analysed by real-time imaging microscope IncuCyte^®^ based on phase-contrast images acquired every 6 h over a period of 120 h (Fig. 4). Neurite length of MN exposed to 400 µM, 600 µM, or 800 µM VX tended to increase initially followed by a decrease. A maximum of neurite length was observed for cells exposed to 400 µM VX after 48 h (76.71 ± 9.71 mm/mm^2^), whereas cells exposed to 600 µM VX revealed a maximum after 24 h (102.06 ± 17.38 mm/mm^2^) and MN exposed to 800 µM VX after 6 h (108.85 ± 15.45 mm/mm^2^). After exposure to 1000 µM VX, neurite length tended to decrease with a minimum of 28.95 ± 9.55 mm/mm^2^ after 72 h. In contrast, a continuous increase in neurite length from 41.67 ± 6.82 mm/mm^2^ (0 h) to 110.25 ± 13.14 mm/mm^2^ (120 h) was detected for MN exposed to solvent control (Fig. 4A). For reasons of comparability, the values of neurite length after 120 h of exposure were normalized to the initial value. Hence, values above 1 represent an increase, while values below 1 indicate a decrease of neurite length. After 120 h of exposure, MN exposed to 400 µM, 600 µM, 800 µM, or 1000 µM VX tended to show no increase in neurite length, while solvent control showed an about 2.6-fold increase (Fig. 4B). Representative phase-contrast images of MN demonstrate a decline in neurite length and branching after 120 h of exposure to all VX concentrations in comparison to solvent control. In MN exposed to 800 µM or 1000 µM VX, a change in phenotype from an organized, branched neuronal network structure to clumped cell aggregates without recognizable cross-linking was recorded, whereas dendrite interactions seemed to remain intact in MN exposed to 400 µM or 600 µM VX (Fig. 4C).

### Differences in gene expression after VX exposure

**Fig. 5:**
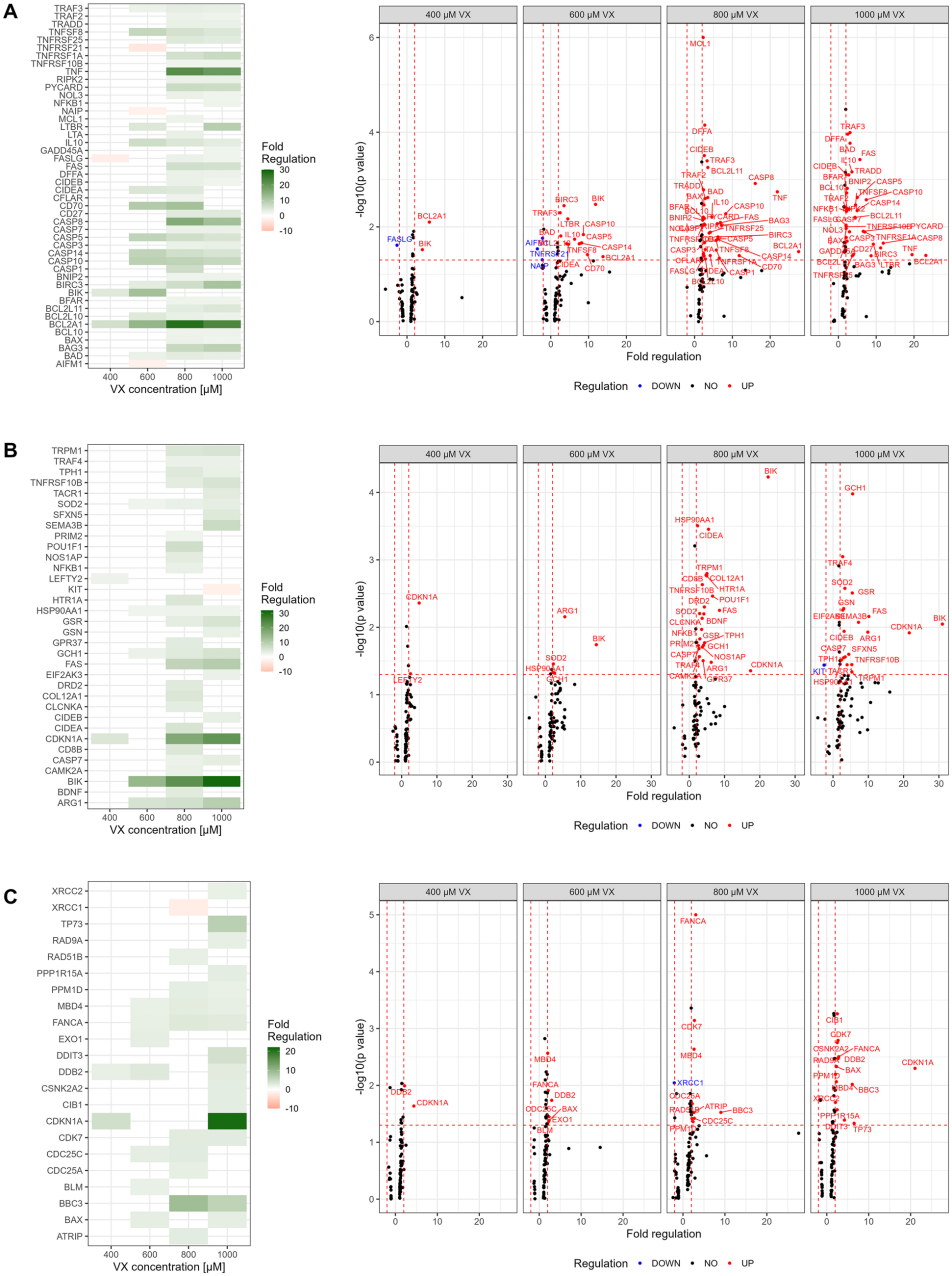
Regulation of genes after VX exposure in MN. Genes associated with apoptosis (**A**), neurotoxicity (**B**) and DNA damage and repair (**C**) were tested 5 days after exposure to 400 µM, 600 µM, 800 µM or 1000 µM VX in comparison to solvent control (ACN) via qRT-PCR. Fold regulation of up- or downregulated genes (≥ 2.0 or ≤  − 2.0 in combination with *p* < 0.05) are shown as means in a heat map (left) and all genes in a volcano plot (right) (*n* = three independent experiments)

After hiPSC-derived MN were exposed to 400 µM, 600 µM, 800 µM, or 1000 µM VX for 120 h, and changes in gene expression in comparison to solvent control were evaluated using established arrays featuring genes involved in apoptosis (Fig. 5A), neurotoxicity (Fig. 5B), and DNA damage and repair (Fig. 5C). The threshold for regulated genes was set as fold regulation ≥ 2.0 or ≤ -2.0 in combination with *p* < 0.05 (Elmore 2007; D’Arcy 2019; Slotkin and Seidler 2012).

Members of the pro-apoptotic gene family ‘cell death-inducing DFFA (DNA fragmentation factor-α)-like effectors (CIDEs)’ such as ‘Cell death inducing DFFA like effector A (CIDEA)’, ‘Cell death-inducing DFFA like effector B (CIDEB)’ or ‘DNA fragmentation factor-α (DFFA, also DFF45)’ (Inohara et al. 1998) were found to be 2- to fivefold upregulated after exposure to 600 µM VX or higher VX concentrations. At similar concentrations, genes involved in neurotoxicity, e.g., superoxide scavenger ‘Superoxide dismutase 2 (SOD2)’, ‘GTP cyclohydrolase 1 (GCH1)’, stress inducible ‘Heat shock protein 90 alpha family class A member 1 (HSP90AA1)’ (Bohush et al. 2019), and nitric oxide response related ‘Arginase 1 (ARG1)’ showed between 2- and tenfold upregulation. Furthermore, a 2- to 22-fold increase in expression was observed for genes related to DNA damage and repair like ‘Cyclin-dependent kinase inhibitor 1A (CDKN1A)’, ‘Cell division cycle 25C (CDC25C)’ (Liu et al. 2020), ‘Damage-specific DNA binding protein 2 (DDB2)’, ‘Methyl-CpG binding domain 4 (MBD4)’, and ‘FA Complementation Group A (FANCA)’.

Mechanisms of cellular stress and DNA damage and repair are known to be stimuli for the activation of the intrinsic pathway of apoptosis. In this context, expressions of pro-apoptotic genes of the BCL-2 family, e.g., ‘BCL2 binding component 3 (BBC3, also PUMA)’, ‘BCL2 associated agonist of cell death (BAD)’, and ‘BCL2 associated X apoptosis regulator (BAX)’ (Hollville et al. 2019), as well as the anti-apoptotic gene ‘BCL2 like 10 (BCL2L10)’, were detected 2- to ninefold increased after exposure to 600 µM VX or higher concentrations. Furthermore, 4- to 12-fold upregulation was recorded for anti-apoptotic gene ‘Baculoviral IAP Repeat Containing 3 (BIRC3, also cIAP2) and anti-inflammatory ‘Interleukin 10 (IL-10)’. ‘Cyclin-dependent kinase 7 (CDK7)’ and ‘BCL2 Interacting Killer (BIK)’, both promotors of apoptosis (Morishita et al. 2013) as well as initiator caspases like ‘Caspase-8 (CASP8)’ or ‘Caspase-10 (CASP10)’, effector caspases e.g., ‘Caspase-3 (CASP3)’ or ‘Caspase-7 (CASP7)’, ‘Caspase-14 (CASP14)’, and ‘Caspase-5 (CASP5)’ associated with inflammation (Elmore 2007) were found to be 3- to 13-fold upregulated.

For genes characteristic of the extrinsic pathway of apoptosis, known as death receptor signalling pathway, like ‘Tumor necrosis factor (TNF)’, ‘Fas cell surface death receptor (FAS, also CD95)’, ‘Fas ligand (FASLG, also CD95L)’, ‘TNF superfamily member 8 (TNFSF8)’, ‘TNFRSF1A associated via death domain (TRADD)’, ‘TNF receptor associated factor 2 (TRAF2)’, ‘Tumor necrosis factor receptor superfamily member 1A (TNFRSF1A, also TNFR1)’, ‘Tumor necrosis factor receptor superfamily member 10b (TNFRSF10B, also TRAIL-R2)’, and ‘Tumor necrosis factor receptor superfamily member 25 (TNFRSF25)’ located at the cell plasma membrane, a 2- to 22-fold increase in expression was detected after exposure to 800 µM or 1000 µM VX (Redza-Dutordoir and Averill-Bates 2016). In this context, TNFRSF25 is linked to promote ‘Nuclear factor of kappa light polypeptide gene enhancer in B-cells 1 (NFKB1)’ activation (Teocchi and D’Souza-Li 2016) which was found 2- to threefold upregulated.

Interestingly, after exposure to 400 µM VX, increased expression was observed only for ‘BCL2 Related Protein A1 (BCL2A1)’, ‘Left–right determination factor 2 (LEFTY)’, CDKN1A, BIK, and DDB2, in between 2- and 28-fold upregulation. Thereby, BCL2A1, represents the only gene that showed a concentration-dependent increase in expression after exposure to 400 µM VX or higher VX concentrations.

Downregulation between 2- and fourfold was observed for genes like ‘KIT proto-oncogene, receptor tyrosine kinase (KIT)’, ‘TNF receptor superfamily member 21 (TNFRSF21)’, ‘NLR family apoptosis inhibitory protein (NAIP, also BIRC1)’, ‘Apoptosis inducing factor mitochondria associated 1 (AIFM1)’, and ‘X-ray repair cross complementing 1 (XRCC1)’. FASLG was the only gene that was threefold downregulated after exposure to 400 µM VX and showed up to threefold upregulation after exposure to 800 µM or 1000 µM VX.

### Apoptotic effects detected by Western blot

**Fig. 6:**
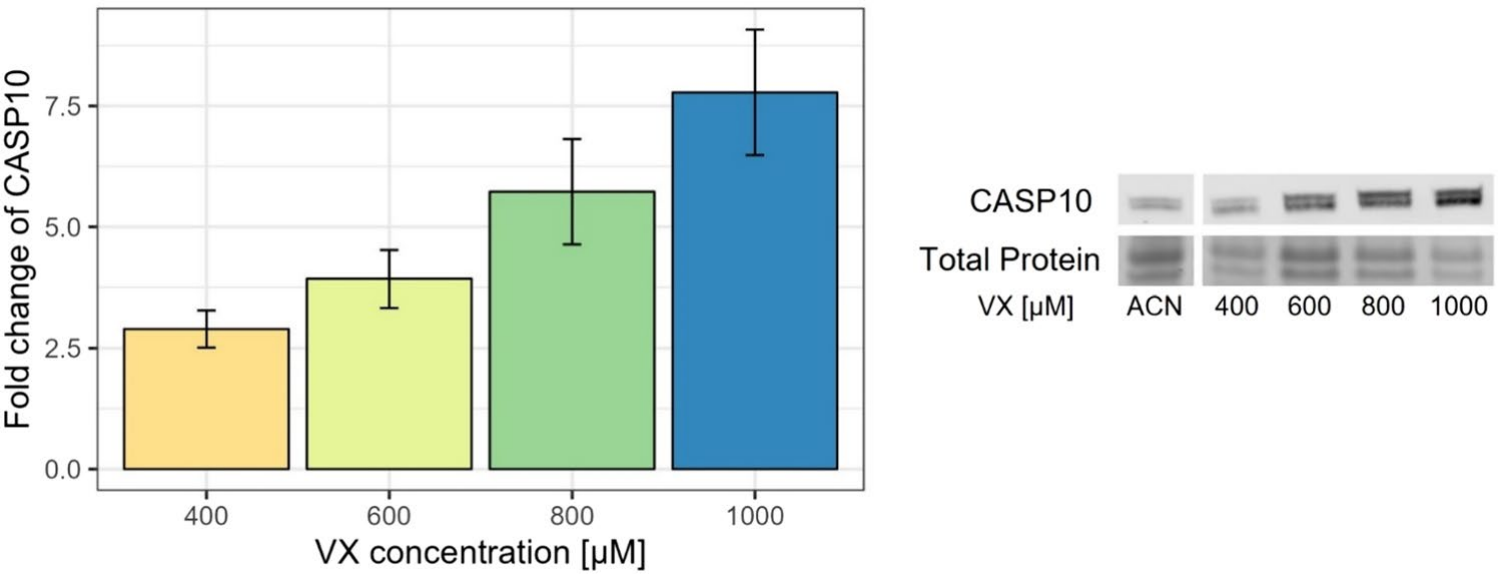
Changes on the protein level in MN. Upregulation of CASP10 in comparison to solvent control (ACN) was observed using Western Blot. Fold changes ± standard deviation and representative bands are shown (*n* = three independent experiments)

To further characterize the effects on MN after exposure to 400 µM, 600 µM, 800 µM, or 1000 µM VX on the protein level, the expression of caspase 10 (CASP10), upregulated on the mRNA level and known to be involved in apoptotic processes, was analysed by Western Blot. CASP10 was upregulated in a concentration-dependent manner in cells after exposure to 400 µM (2.90 ± 0.38), 600 µM (3.93 ± 0.60), 800 µM (5.73 ± 1.09), or 1000 µM VX (7.78 ± 1.30) compared to solvent control (Fig. 6). These findings correlate with the qRT-PCR results (Fig. 5A) showing upregulation after exposure to 600 µM VX or higher VX concentrations.

## Discussion

In this study, the effects of VX on hiPSC-derived MN were investigated for the first time which provides new insight into the non-cholinergic mechanism after VX exposure in MN as part of the human neuromuscular junction. Non-cholinergic mechanisms are described to be affected by OPC like VX, when non-AChE-enzymes and proteins e.g., receptor channel complexes, are chemically modified and their functionality is altered (John et al. 2020). These chemical modifications require high OPC concentrations that exceed the lethal dose, which limits their relevance in acute poisoning scenarios (John et al. 2020). Nevertheless, it can be assumed that intoxications with high VX concentrations, such as those used here, could be the scenario of a terrorist attack. Furthermore, specific cell types, e.g., mesenchymal stem cells showed a high tolerance to chemical ware fare agents such as sulfur mustard (Schmidt et al. 2013). This underlines the need to evaluate the sensitivity of hiPSC-derived MN to VX as a prerequisite for integration in a human neuromuscular test model.

For reasons of translation of the results, MN used in this study were differentiated from in-house feeder- and integration-free-derived hiPSC by application of antibiotic-free and standardized differentiation media with the aim to closely mimic human embryogenesis. MN were regularly tested for state-specific neuronal markers (Schaefers et al. 2022), ensuring the type and quality of cells being used for the experiments.

After single-dose exposure with 400 µM or 1000 µM VX following incubation for 5 days and analysis of MN proteins via µLC-ESI MS/HR MS (PIS), a concentration-dependent phosphonylation of tyrosine residues was detected, demonstrating the interaction of VX with cellular proteins (Fig. 1). Notably, dose-dependent interaction with tyrosine residues has been reported before in neuronal proteins such as ß-tubulin after OPC exposure, suggesting an impairment of microtubule function and polymerization resulting in synaptic degradation (Terry 2012; Hasmik Grigoryan et al. 2009). The determination of VX-adducts with specific proteins, e.g., ß-tubulin, is part of current studies. To further investigate the concentration-dependent effects, MN were additionally exposed to 600 µM or 800 µM VX. After 5 days of exposure to 400 µM, 600 µM, 800 µM, or 1000 µM VX, significant cytotoxic effects analysed by XTT were observed only after exposure to 1000 µM VX, whereas exposure to 400 µM VX resulted in an increased viability compared to solvent control (Fig. 2A). In comparison, cellular growth recorded by the IncuCyte^®^ was significantly decreased after exposure to all VX concentrations compared to solvent control. Even though, MN exposed to 400 µM VX tended to show an increase in cellular growth normalized to the initial value before VX exposure (Fig. 3B). The difference in results of the XTT and the IncuCyte^®^ testing method might be due to the downregulation of glycolysis during NPC differentiation into MN which might cause a lack of NADH (Maffezzini et al. 2020; Zheng et al. 2016) being essential for the reduction of XTT to detectable formazan (Berridge et al. 2005; McGaw et al. 2014). This could also be an explanation for the high variations in the XTT results of MN compared to VX-exposed NPC (Fig. 2B). Thus, the XTT‑assay seems to be applicable only for investigation of effects on NPC. In contrast, glycolysis represents no decisive parameter in the analysis by the IncuCyte^®^ and, therefore, might be more appropriate for the detection of VX related changes in MN.

Interestingly, during 5 days of VX exposure, a decrease in confluence was detected at different time points in addition to the different VX concentrations, indicating a time and concentration dependence (Fig. 3A) which was reported previously after exposure to OPC (Liu et al. 2019). Similarly, neurite length also seemed to be altered in a time- and concentration-dependent manner but tended to decrease even earlier (Fig. 4A). These time- and concentration-dependent changes in neurite outgrowth preceding cell death are in line with the previous publications on neurons exposed to OPC (Hong et al. 2003; Christen et al. 2017; Carlson and Ehrich 2001). Additionally, the changes in morphology, e.g., the formation of fragments resembling apoptotic bodies and the number of detached cell aggregates, seemed to increase concentration-dependently after VX exposure. Thus, potentially viable cells with intact cell membrane and dendrite network remained after exposure to 400 µM or 600 µM VX, whereas the morphology of MN exposed to 800 µM or 1000 µM VX changed dramatically as cell shrinkage and neurite loss dominated (Fig. 3C, Fig. 4C). These effects on morphology and neurite outgrowth are consistent with findings of Yousefpour et al. (2006) revealing dose-dependent induction of neurotoxicity and neuronal cell death following treatment with OPC paraoxon.

The mechanism underlying cell death and neurotoxicity after VX exposure is not yet fully understood. Gao et al. suggest that genes responsible for basic cellular functions (e.g., DNA replication) might be affected or genes related to DNA damage and apoptosis might be induced after VX exposure (Gao et al. 2013). Moreover, mitochondrial dysfunction and reactive oxygen species (ROS) are described to be involved in cell death after OPC exposure (Lopez-Suarez et al. 2022; Turton et al. 2021; Tallat et al. 2020; Hilgert Jacobsen-Pereira et al. 2018). In correlation, results of gene expression analysis of this study (Fig. 5) indicate an upregulation after exposure to 600 µM VX or higher concentrations of genes of the CIDE family (e.g., CIDEA, CIDEB, DFFA), which are associated with the induction of DNA fragmentation and an morphology which is characteristic of apoptosis (Inohara et al. 1998), as well as of genes related to DNA damage and repair (e.g., CDC25C, MBD4, FANCA) (Liu et al. 2020; Benitez et al. 2018; Dinis et al. 2012). DNA damage response in neurons is associated with the induction of genes characteristic for the intrinsic pathway of apoptosis such as pro-apoptotic PUMA and BAX (Shadfar et al. 2022; Wyttenbach and Tolkovsky 2006). These were found to be upregulated in MN exposed to 600 µM VX or higher VX concentrations and support the formation of pores into the mitochondrial membrane resulting in mitochondrial outer membrane permeabilization (MOMP) (Steckley et al. 2007). In mammalian cells, pore formation is described to rely on oligomerization of BCL2 Antagonist/Killer 1 (BAK) and BAX for induction of apoptosis. However, in neurons, induction of apoptosis was shown to be only dependent on BAX expression (Wright and Deshmukh 2006) which may coincide with the lack of BAK upregulation observed in this study. Subsequently, effector caspases such as CASP3 and CASP7, upregulated after exposure to 800 µM or 1000 µM VX, might be activated, thus resulting in apoptotic cell death.

Interestingly, neuroprotective mechanisms in response to inflammatory events seemed to be triggered after exposure to 600 µM VX or higher VX concentrations as genes like ARG1, SOD2, and IL10 were upregulated. These are known to be involved in various neurodegenerative diseases associated with neuroinflammation e.g., multiple sclerosis, amyotrophic lateral sclerosis (ALS), Alzheimer’s or Parkinson’s disease (Lewis et al. 2014; Liu et al. 2022; Porro et al. 2020; Ma et al. 2020). Strikingly, Alzheimer’s and Parkinson’s disease were suggested previously to be a consequence of VX-induced changes in gene expression (Gao et al. 2013).

After exposure to 800 µM or 1000 µM VX, pro-apoptotic mechanisms seemed to dominate as several genes coding for death receptors and ligands, such as a subset of tumor necrosis factor (TNF) superfamily (e.g., TNF, TNFR1, TNFRSF10B, TNFRSF25) associated with initiation of the extrinsic pathway of apoptosis (Redza-Dutordoir and Averill-Bates 2016), were found to be upregulated. The formation of death-inducing signal complex (DISC), a key event in the extrinsic pathway of apoptosis (Dickens et al. 2012), requires interaction of FASLG, FAS, TNF, TNFR1, TRAF2 and among others TRADD (Elmore 2007; Tummers and Green 2017; Guidotti et al. 2021) that were upregulated after VX exposure. In line with this finding, TNF and FAS were already suggested to be involved in OPC-induced apoptosis (Carlson et al. 2000). Subsequent activation of initiator caspases, such as CASP8 or CASP10, might further trigger pro-apoptotic proteins like BAX leading to a crosstalk with the intrinsic pathway of apoptosis (Teocchi and D’Souza-Li 2016).

Interestingly, the functional role of CASP10, a close homologue of CASP8, is controversially discussed. Previous studies indicated a pro-apoptotic function for CASP10 in combination with CASP8 in cell death signaling (Mohr et al. 2018; Mühlethaler-Mottet et al. 2011; Wachmann et al. 2010; Wang et al. 2001). However, Horn and Hughes et al. suggested that CASP10 promotes cell survival by switching response to NFKB signaling (Horn et al. 2017). This assumption might be in accordance with the results of this study as CASP10 expression in protein analysis was increased after exposure to 400 µM VX (Fig. 6), but upregulation of CASP8 and other pro-apoptotic genes (e.g., CASP3, CASP7, TNFRs) as well as the loss in confluence were detected mainly after exposure to 800 µM VX or higher VX concentrations.

Furthermore, BIK, often reported as a promotor of apoptosis and to be increased after exposure to 400 µM VX or higher VX concentrations, was found to induce an inflammatory, anti-apoptotic response in co-expression with CASP10 (Chinnadurai et al. 2008; Han et al. 1996). Anti-apoptotic effects might be further enhanced by increased expression of CDKN1A and DDB2. These are induced in response to DNA damage (Rikhof et al. 2003; Li et al. 2012) and described to cooperate in induction of premature senescence (Roy et al. 2012), a stress-related form of cell growth arrest that is usually resistant to apoptosis, accompanied by morphological alterations and triggered by exposure to sub-cytotoxic agents (Kumari and Jat 2021; Petrova et al. 2016). Although neuronal senescence remains largely unexplored (Shadfar et al. 2022), selective upregulation of LEFTY2, known as an inhibitor of cell growth (Sun et al. 2014), in combination with the senescence-specific enlarged, flattened morphology of MN exposed to 400 µM VX might strengthen this assumption. For clarification, the investigation of senescent markers is part of future research.

Strikingly, BCL2A1 was the only gene upregulated concentration-dependently after exposure to 400 µM VX or higher VX concentrations. With progression of neuroinflammation, increasing BCL2A1 expression is assumed to promote loss of MN, thus switching to a pro-apoptotic role (Crosio et al. 2006). This might stand in line with results of this study as increased expression of pro-apoptotic genes dominated in MN after exposure to 800 µM or 1000 µM VX.

In summary, for the first time, this study demonstrates that VX induces time- and concentration-dependent neuronal cell death in hiPSC-derived MN. Upon exposure to 600 µM VX or higher VX concentrations, neuroinflammatory mechanisms, the intrinsic as well as the extrinsic pathway of apoptosis seemed to be activated coinciding with the accumulation of cell fragments resembling apoptotic bodies and decrease of neurite length. In contrast, after exposure to 400 µM VX, potentially viable MN with an altered morphology and dendrite network exhibited increased expression of genes characteristic for DNA damage response, thus suggesting the induction of premature senescence. As the hiPSC-derived MN used in this study were sensitive to VX exposure, they may constitute a promising tool for the development of a neuromuscular test model. To accomplish this aim, it is essential to test hiPSC-derived skeletal muscle cells to VX sensitivity and bring them into functional cooperation with hiPSC-derived MN. This model may allow the investigation of poisoning with structurally related, less-toxic OP pesticides or motoneuronal degenerative diseases, e.g., ALS, in the future.
